# A Simple Method for Compensating Harmonic Distortion in Current Transformers: Experimental Validation

**DOI:** 10.3390/s21092907

**Published:** 2021-04-21

**Authors:** Christian Laurano, Sergio Toscani, Michele Zanoni

**Affiliations:** 1 Ricerca sul Sistema Energetico S.p.A., via Rubattino 54, 20134 Milano, Italy; christian.laurano@rse-web.it (C.L.); michele.zanoni@rse-web.it (M.Z.); 2 Politecnico di Milano—DEIB, Piazza Leonardo da Vinci 32, 20133 Milano, Italy

**Keywords:** error compensation, harmonic distortion, instrument transformers, current transformers, current measurement, calibration, nonlinear systems, frequency response, power system harmonics, measurement uncertainty, frequency domain analysis

## Abstract

Conventional current transformers (CTs) suffer from nonlinearities due to their ferromagnetic cores. On one hand, it is well-known that severe core saturation may occur because of large overcurrents or unidirectional transient components: this may substantially impact the operation of relays. On the other hand, weaker nonlinear effects are also present during regular working conditions. In particular, the spectral content of typical current waveforms is characterized by a strong fundamental term responsible for harmonic distortion affecting the frequency components at the secondary side. In turn, this has a significant impact on the accuracy that can be reached as long as current harmonics must be monitored. The target of this work is implementing a simple signal processing technique that allows compensating for this effect. The method, characterized by extremely low computational complexity, is first introduced and validated using numerical simulations. After this, it was tested experimentally to improve the harmonic measurement capability of inductive CTs. The achieved results highlight a noticeable reduction of errors at low-order harmonics over a wide range of primary current amplitudes. It is worth noting that the black-box approach makes the technique suitable also for compensating nonlinearities introduced by current transducers based on different operating principles. Thanks to this peculiarity and to the low computational complexity, the proposed method is suitable to be employed in power quality analyzers and merging units. In this way, high-accuracy monitoring of current harmonics in power systems can be achieved, opening the way to advanced power quality management and to the location of disturbing users.

## 1. Introduction

Although many current transducers based on different operating principles are available on the market, conventional inductive current transformers (CTs) are still extensively employed to measure currents in AC power systems, both for relaying and metering purposes. Their simple yet effective operating principle represents the main reason: it ensures galvanic insulation between the primary and secondary sides, and it permits measuring the fundamental component with adequate accuracy for most of the applications. Furthermore, since they may be subject to harsh environmental conditions, their excellent long-term performance stability and exemplary reliability represent two of their most relevant advantages. Unfortunately, conventional CTs also suffer from several drawbacks; it is worth noting that most of them are strictly related to the iron core that ensures tight magnetic coupling between primary and secondary circuits and that enables the previously described features.

The most noticeable issue related to core nonlinearity occurs during faults and switching. The resulting large overcurrents and slowly decaying transient terms produce a consistent increase of core flux density so that the core material may operate near or even above its knee-point on the magnetization characteristic. This may produce a very significant magnetizing current that, in turn, jeopardizes the secondary output, which does not provide an accurate representation of what is happening at the primary side. When the CT is employed for protection, this may result in a delayed intervention of overcurrent relays, false tripping of differential relays, wrong impedance calculation in directional relays; all of these effects have a substantial impact on grid operation. It is no surprise that the core saturation of CTs and their effects have been studied for a long time. The obvious method to mitigate this phenomenon is using a CT with a larger core cross-section and secondary winding area, but this translates into increased size and weight and higher manufacturing cost. Another possibility is applying proper processing techniques to the secondary current signal to detect the occurrence of saturation [1] and possibly reducing its impact [2,3,4]. In this respect, [2] employs the equations of the usual equivalent circuit model of the CT (with single-valued, polynomial magnetizing current-flux characteristics) to reconstruct the primary current from the measured secondary current, thus compensating nonlinear effects; preliminary model identification is required, and a least-squares (LS) approach is adopted. The technique proposed by [3] is based on a completely different idea: CT saturation that may occur during faults is mitigated through LS identification of a simple parametric model of the primary current waveform (a sinewave with a superimposed decaying exponential) from the measured one. [4] compares two approaches to retrieve the primary current signal: an LS fitting method (in principle similar to that presented in [3]) and a technique based on artificial neural networks.

Measuring current and voltage harmonics represents a very important task for power quality assessment [5] in power systems: in conjunction with proper techniques, it permits studying the propagation of harmonic disturbances on the grid and identifying their cause [6,7]. Even more advanced methods can be implemented if these measurements in remote nodes of the grid are performed in a shared timescale: in fact, the study of harmonic phasor measurement units and their applications [8] are popular research topics in recent years. In this respect, the effectiveness of the aforementioned techniques strictly depends on the accuracy of the available measurements, which in turn are heavily affected by the characteristics of the employed transducers [9].

The conventional CTs installed in the network are often used to perform the harmonic current measurement, although their metrological performance is quantified by their accuracy class, which provides reliable information only when the sole fundamental component must be measured [10]. Accuracy requirements for harmonic measurements are specified only as far as low-power instrument transformers (LPITs) are concerned [11]. On the other hand, the impact of conventional CTs on harmonic measurements has been investigated by scientific papers for many years [12,13,14,15]; all of them agree that, in general, harmonic amplitudes and phases are transduced with considerably worse accuracy in comparison with the fundamental term. The main reason is often supposed to be the bandwidth limitation [16] due to the dynamics of the CT; techniques aimed at compensating its frequency response can be found in the literature [17,18,19]. However, (weak) nonlinearities are also present even when the core operates well below saturation [20,21]; their impact is significant as long as the measurement of low-order harmonics is concerned. In principle, they can be mitigated through a specific design of the CT [20] or by adopting signal processing techniques that enable significantly more accurate harmonic measurements. In this respect, [22] proposes to compensate for nonlinearities through frequency-domain linearization around a specific working condition, defined by the amplitude of the fundamental component.

Similar to the case of CTs, also the metrological performance of conventional voltage transformers (VTs) employed for harmonic measurement is strictly related to their dynamic and nonlinear behavior [23]. In this respect, the authors of the present paper have shown that nonlinear behavioral models based on a simplified Volterra representation [24,25] can be used to accurately predict the secondary voltage spectrum from the primary side [26]. This enables a thorough characterization of VTs: linear and nonlinear effects can be separated, nonlinearities can be classified, and their individual impact on the overall metrological performance can be studied. Furthermore, identifying the inverse model permits a very precise reconstruction of the primary voltage harmonics from the secondary side, thus compensating most of the nonlinearities other than the filtering behavior [27].

The key assumption that permits formulating and effectively applying the aforementioned models is that the input quantity is quasi-sinusoidal, thus consisting of a strong fundamental component superimposed to harmonics, which, one-by-one, have considerably lower amplitudes, as typically occurs in voltage waveforms. A further simplification can be obtained by observing that, under these conditions, the strongest nonlinear effect is the harmonic distortion (HD) produced by the large fundamental term. From a different point of view, it corresponds to neglect the interactions between fundamental and harmonics: this permits developing very simple techniques [28,29] that allow retrieving the primary voltage spectrum from the secondary side by considering the effect of HD. In particular, the method proposed in [28] is based on polynomial modeling and compensation of the HD. One of its main advantages lies in model structure, which permits adopting an LS-based preliminary identification procedure. Therefore, it does not require applying a specific set of primary waveforms, but a broad class of realistic periodic voltages resulting in a determined or overdetermined problem can be employed. This results in higher flexibility and, most importantly, weak constraints about the metrological characteristics of the generation system that allows applying such waveforms.

The remarkable results suggest testing the same technique to improve the harmonic measurement accuracy that can be obtained with CTs; however, major differences arise in this case. The first one is that current waveforms typically have higher harmonic content than voltage waveforms. In addition, a key hypothesis that has been exploited in [28] when deriving the polynomial HD compensation method is the linear behavior at the fundamental component. This assumption is met in VTs [23], but it is somewhat weaker in CTs since a considerably wider measuring range is required. From a different perspective, it is easier to obtain a simple yet accurate model for a reduced input range; when it is extended, performance may significantly degrade. In this respect, a preliminary study about the accuracy that can be reached by applying HD compensation to CTs is carried out in [30] using numerical simulations; the promising results suggest performing further theoretical investigations aimed at the experimental application, which is the target of the present paper.

The first step was adapting the polynomial HD compensation technique to CTs, and in particular, the linearity assumption about the fundamental component was removed. After this, the impact of considering a linear relationship between primary and secondary side fundamental current was assessed through numerical simulations, which confirm the validity of the assumption: it enables a remarkable simplification of the method without significant drawbacks in terms of accuracy. Finally, the technique was experimentally implemented through a proper setup and employed to improve the harmonic measurement performance of two different inductive CTs characterized by different rated accuracy; for a deeper understating about the potentiality of the HD compensation technique, two classes of primary current waveforms having different distortion levels were applied. The results obtained by applying many primary current waveforms belonging to the aforementioned classes were analyzed thoroughly. They highlight the noticeable accuracy improvement that can be reached thanks to the proposed method, other than confirming that the accuracy class of a CT is not representative of the harmonic measurement performance.

## 2. Harmonic Distortion Compensation Technique

Let us consider a CT operating in a power system having rated frequency *f*_0_, thus corresponding to the angular frequency *ω*_0_. The primary current waveform *i*_1_ is supposed to be a periodic multisine having rated frequency and containing a fundamental component that is considerably higher concerning the other harmonics (quasi-sinusoidal assumption), as typically happens during normal operation. The CT can be considered as a nonlinear time-invariant system; therefore, excluding complex nonlinearities (such as chaos and limiting cycles) also the secondary current *i*_2_ is a periodic multisine waveform characterized by the same fundamental frequency *f*_0_. Since the study will be carried out in the frequency-domain, it is worth introducing the generic *m*th order harmonic component at the primary and secondary side; they will be indicated as *I*_1_(*m*) and *I*_2_(*m*), respectively. *I*_2_(*m*) can be decomposed as follows:

(1) $$\begin{array}{c} I_{2}\left(m\right)=I_{2,L}\left(m\right)+I_{2,NL}\left(m\right)\\ I_{2,L}\left(m\right)=H_{1}\left(m\right)I_{1}\left(m\right) \end{array}$$

*I*_2,*L*_(*m*) is proportional to the primary current harmonic having the same order, namely *I*_1_(*m*), through the complex-valued coefficient *H*_1_(*m*). It would be the only contribution if the CT were characterized by a perfectly linear behavior. On the other hand, *I*_2,*NL*_(*m*) allows taking into account nonlinearities: in general, it is a function of all the primary current harmonics. When required, a third contribution could be included to model the impact of additive noise.

### 2.1. Polynomial Representation of HD

As aforementioned, the fundamental term has the largest magnitude among the primary current spectral components. Because of this peculiarity, the strongest nonlinear effect produced by the CT is the HD due to the strong fundamental primary current, while interactions between different spectral components are expected to have a significantly weaker impact on the secondary side; therefore, they can be neglected in first approximation. Under this assumption, *I*_2,*NL*_(*m*) is the result of HD, and hence it is a function *f_m_* of the fundamental primary current *I*_1_(1) that changes according to the considered secondary side harmonic order *m*. Therefore, (1) can be written as:

(2) $$I_{2}\left(m\right)\approx H_{1}\left(m\right)I_{1}\left(m\right)+f_{m}\left(I_{1}\left(1\right)\right)$$

In turn, a polynomial approach [31] is adopted to obtain a model that allows considering the effect of HD. After some manipulations, assuming *m* ≥ 2, *f_m_* results:

(3) $$\begin{array}{c} f_{m}\left(I_{1}\left(1\right)\right)=\sum_{l=0}^{\infty }H_{m+2l}\left(m\right){\left|I_{1}\left(1\right)\right|}^{m+2l}e^{jm\varphi_{1}}\\ \varphi_{1}=∠I_{1}\left(1\right) \end{array}$$

Considering the generic *m*th order harmonic in the secondary current, it results from theoretically infinite contributions, each one characterized by its order or degree *d* = *m* + 2*l*. When *m* = 1, which is the impact on the secondary-side fundamental term, the summation in (3) must be lower-bounded to 1. It is easy to notice that, according to this model, the *m*th order secondary harmonic is unaffected by contributions whose degrees *d* are lower than *m*. Furthermore, only even degree contributions influence even order harmonics, while odd order harmonics are exclusively affected by odd degree contributions. When (3) is substituted into (2), we get the expression of the generic secondary current harmonic (*m* ≥ 2):

(4) $$I_{2}\left(m\right)\approx H_{L}\left(m\right)I_{1}\left(m\right)+\sum_{l=0}^{\infty }H_{m+2l}\left(m\right){\left|I_{1}\left(1\right)\right|}^{m+2l}e^{jm\varphi_{1}}$$

In (3) and (4), harmonic distortion is modeled by an infinite number of terms: this prevents the practical implementation. For this reason, a maximum value for the nonlinearity degree *d* is considered. This corresponds to add the following constraint:

(5) m+2l≤D

where *D* is the degree (or order) of the polynomial representation. Bearing in mind the previous considerations, it is worth noting that a *D*th degree model is not capable of taking into account HD contributions to secondary side current harmonics whose orders are greater than *D*. Therefore, for *m* > *D*, the adopted model becomes a frequency response function.

### 2.2. HD Compensation Technique

In the previous subsection, a polynomial model of HD was introduced. Now the target is using it to reconstruct the primary side current spectrum from the secondary side. For this purpose, let us consider (4) with *m* ≥ 2 and a finite degree *D*:

(6) $$I_{2}\left(m\right)\approx H_{L}\left(m\right)I_{1}\left(m\right)+\sum_{l=0}^{\left\lfloor \frac{D-m}{2}\right\rfloor }H_{m+2l}\left(m\right){\left|I_{1}\left(1\right)\right|}^{m+2l}e^{jm\varphi_{1}}$$

where ⌊⋅⌋ denotes the integer part function. When *m* = 1:

(7) $$I_{2}\left(1\right)\approx H_{L}\left(1\right)I_{1}\left(1\right)+\sum_{l=1}^{\left\lfloor \frac{D-1}{2}\right\rfloor }H_{1+2l}\left(1\right){\left|I_{1}\left(1\right)\right|}^{1+2l}e^{j\varphi_{1}}$$

A notable feature is that, according to this representation, the behavior at the fundamental component is not affected by the presence of other harmonics in the primary current. This peculiarity is often exploited during the study of conventional instrument transformers; it is confirmed by the experimental results presented in many works and specifically addressed by [32]. (6) can be rewritten as:

(8) $$I_{1,e}\left(m\right)=\frac{I_{2}\left(m\right)-\sum_{l=0}^{\left\lfloor \frac{D-m}{2}\right\rfloor }H_{m+2l}\left(m\right){\left|I_{1}\left(1\right)\right|}^{m+2l}e^{jm\varphi_{1}}}{H_{L}\left(m\right)}$$

According to the previous formula, to compute *I*_1,*e*_(*m*), which is the estimate of the generic *m*th order primary side harmonic, the coefficients *H_i_*(*m*), *H_L_*(*m*) as well as an estimate of the fundamental primary current *I*_1_(1) are required.

Let us apply a proper set of *P* quasi-sinusoidal, periodic currents to the primary winding of the CT so that we are under the assumptions that the proposed approach is based on; the generic *p*th waveform (*p*∈{1,..,*P*}) is characterized by known primary harmonics *I*_1_^[*p*]^(*m*) that define its spectrum. Let us observe the corresponding secondary current harmonics *I*_2_^[*p*]^(*m*), which can be arranged in column vectors **I**_2_(*m*). For each test realization, (6) allows obtaining an estimate *I*_2,*e*_^[*p*]^(*m*) of the generic secondary current harmonic. Considering all the *P* excitations while using matrix notation leads to:

(9) $$\left[\begin{array}{c} I_{2,e}^{\left[1\right]}\left(m\right)\\ I_{2,e}^{\left[2\right]}\left(m\right)\\ \vdots \\ I_{2,e}^{\left[P\right]}\left(m\right) \end{array}\right]=\left[\begin{array}{cccc} I_{1}^{\left[1\right]}\left(m\right) & {\left|I_{1}^{\left[1\right]}\left(1\right)\right|}^{m}e^{jm\varphi_{1}} & {\left|I_{1}^{\left[1\right]}\left(1\right)\right|}^{m+2}e^{jm\varphi_{1}} & \cdots \\ I_{1}^{\left[2\right]}\left(m\right) & {\left|I_{1}^{\left[2\right]}\left(1\right)\right|}^{m}e^{jm\varphi_{1}} & {\left|I_{1}^{\left[2\right]}\left(1\right)\right|}^{m+2}e^{jm\varphi_{1}} & \cdots \\ \vdots & \vdots & \vdots & \ddots \\ I_{1}^{\left[P\right]}\left(m\right) & {\left|I_{1}^{\left[P\right]}\left(1\right)\right|}^{m}e^{jm\varphi_{1}} & {\left|I_{1}^{\left[P\right]}\left(1\right)\right|}^{m+2}e^{jm\varphi_{1}} & \cdots \end{array}\right]\left[\begin{array}{c} H_{L}\left(m\right)\\ H_{m}\left(m\right)\\ H_{m+2}\left(m\right)\\ \vdots \end{array}\right]\;\begin{array}{c} I_{2,e}\left(m\right)=W_{1,m}H_{m} \end{array}$$

when *m* = 1, (7) must be used. Following the same passages:

(10) $$\left[\begin{array}{c} I_{2,e}^{\left[1\right]}\left(1\right)\\ I_{2,e}^{\left[2\right]}\left(1\right)\\ \vdots \\ I_{2,e}^{\left[P\right]}\left(1\right) \end{array}\right]=\left[\begin{array}{cccc} I_{1}^{\left[1\right]}\left(1\right) & {\left|I_{1}^{\left[1\right]}\left(1\right)\right|}^{3}e^{j\varphi_{1}} & {\left|I_{1}^{\left[1\right]}\left(1\right)\right|}^{5}e^{j\varphi_{1}} & \cdots \\ I_{1}^{\left[2\right]}\left(1\right) & {\left|I_{1}^{\left[2\right]}\left(1\right)\right|}^{3}e^{j\varphi_{1}} & {\left|I_{1}^{\left[2\right]}\left(1\right)\right|}^{5}e^{j\varphi_{1}} & \cdots \\ \vdots & \vdots & \vdots & \ddots \\ I_{1}^{\left[P\right]}\left(1\right) & {\left|I_{1}^{\left[P\right]}\left(1\right)\right|}^{3}e^{j\varphi_{1}} & {\left|I_{1}^{\left[P\right]}\left(1\right)\right|}^{5}e^{j\varphi_{1}} & \cdots \end{array}\right]\left[\begin{array}{c} H_{L}\left(1\right)\\ H_{3}\left(1\right)\\ H_{5}\left(1\right)\\ \vdots \end{array}\right]\;\begin{array}{c} I_{2,e}\left(1\right)=W_{1,1}H_{1} \end{array}$$

Assuming that *P* is greater than the length of **H***_m_* and that the applied waveforms result in matrices **W**_1,*m*_ having full column rank, their Moore–Penrose inverse can be computed. In this case, the coefficient vectors **H***_m_* can be estimated with the LS approach. It corresponds to the minimization of the Euclidean norm of the vector **I**_2_(*m*)-**I**_2,*e*_(*m*), whose components are the *P* complex errors between the measured and predicted *m*th order current harmonics at the secondary side. As aforementioned during the introduction, this LS-based identification procedure does not require injecting specific current waveforms for estimating the model parameters; in this respect, the advantage is twofold. First, there is large flexibility in the choice of the identification signals. From another point of view, there is no need for a generation system able to apply these current waveforms with very high accuracy, thus reducing the cost and complexity of the implementation.

However, using (8) for reconstructing the primary current spectrum, requires an estimate *I*_1,*e*_(1) of the fundamental primary current *I*_1_(1). For this purpose, let us consider (7); *I*_2_(1) is known since it can be directly measured. Therefore, it represents a polynomial equation that can be numerically solved to compute *I*_1,*e*_(1), namely an estimate of the corresponding fundamental primary current. Multiple solutions are obtained, but since nonlinearities are expected to have a small impact at the fundamental, the solution closest to *H_L_*^−1^(1)*I*_2_(1) (linear approximation) is considered.

Finally, (8) can be employed to reconstruct the primary current harmonics from the secondary side while taking into account the effect of HD. However, because of the structure of the underlying model, this technique can mitigate HD in harmonics whose orders are equal or lower than *D*. Otherwise, no terms appear in the summation in (6), namely the reconstruction of the primary side harmonics becomes linear.

### 2.3. Simplified Approach

Let us suppose that the relationship between primary and secondary fundamental currents is linear with good accuracy; hence, it is possible to write:

(11) $$I_{1}\left(1\right)\approx K_{L}\left(1\right)I_{2}\left(1\right)$$

Having introduced this additional assumption, a considerable simplification is obtained:

(12) $$\begin{array}{c} I_{1}\left(m\right)\approx I_{1,e}\left(m\right)=K_{L}\left(m\right)I_{2}\left(m\right)+\sum_{l=0}^{\left\lfloor \frac{D-m}{2}\right\rfloor }K_{m+2l}\left(m\right){\left|I_{2}\left(1\right)\right|}^{m+2l}e^{jm\varphi_{2}}\\ \varphi_{2}=∠I_{2}\left(1\right) \end{array}$$

According to (12), obtaining the estimates *I*_1,*e*_(*m*) of the primary current harmonics from the secondary spectrum requires computing the coefficients *K_L_*(*m*) and *K_d_*(*m*). They can be obtained by using the same approach adopted in the previous subsection, which is applying a proper set of *P* quasi-sinusoidal primary currents, observing the corresponding steady-state responses so that it is possible to write:

(13) $$\left[\begin{array}{c} I_{1,e}^{\left[1\right]}\left(m\right)\\ I_{1,e}^{\left[2\right]}\left(m\right)\\ \vdots \\ I_{1,e}^{\left[P\right]}\left(m\right) \end{array}\right]=\left[\begin{array}{cccc} I_{2}^{\left[1\right]}\left(m\right) & {\left|I_{2}^{\left[1\right]}\left(1\right)\right|}^{m}e^{jm\varphi_{2}} & {\left|I_{2}^{\left[1\right]}\left(1\right)\right|}^{m+2}e^{jm\varphi_{2}} & \cdots \\ I_{2}^{\left[2\right]}\left(m\right) & {\left|I_{2}^{\left[2\right]}\left(1\right)\right|}^{m}e^{jm\varphi_{2}} & {\left|I_{2}^{\left[2\right]}\left(1\right)\right|}^{m+2}e^{jm\varphi_{2}} & \cdots \\ \vdots & \vdots & \vdots & \ddots \\ I_{2}^{\left[P\right]}\left(m\right) & {\left|I_{2}^{\left[P\right]}\left(1\right)\right|}^{m}e^{jm\varphi_{2}} & {\left|I_{2}^{\left[P\right]}\left(1\right)\right|}^{m+2}e^{jm\varphi_{2}} & \cdots \end{array}\right]\left[\begin{array}{c} K_{L}\left(m\right)\\ K_{m}\left(m\right)\\ K_{m+2}\left(m\right)\\ \vdots \end{array}\right]\;\begin{array}{c} I_{1,e}\left(m\right)=W_{2,m}K_{m} \end{array}$$

As usual, *I*_1_^[*p*]^(*m*) and *I*_2_^[*p*]^(*m*) with *p*∈{1,..,*P*} are the values of the generic primary and secondary *m*th order harmonic corresponding to the *p*th test. Having supposed that the matrices **W**_2,*m*_ have full column rank, vectors **K***_m_* are estimated in the LS sense.

## 3. Numerical Simulations

The first part of this work aims to compare the two proposed HD compensation methods to select the best one for practical applications, namely that ensuring the best tradeoff between accuracy and performance. The study will be carried out through numerical simulations by considering a detailed model of an inductive CT, which is capable of taking into account the hysteretic, nonlinear behavior of its core. In this way, the comparison results are not affected by noise and measurement uncertainty that may mask minor differences.

### 3.1. Reference Model of the CT

**Figure 1:**
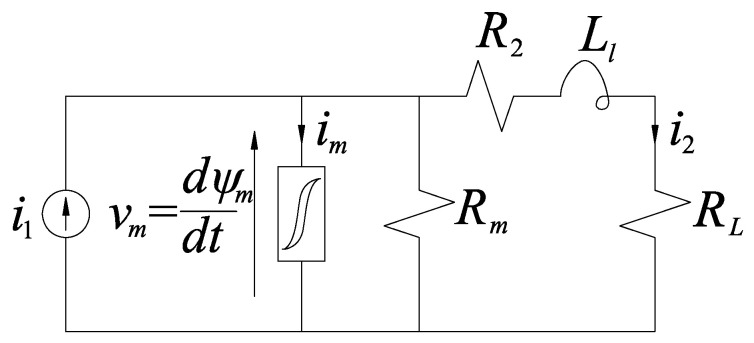
Equivalent circuit of the CT.

When considering frequencies up to few kilohertz, a conventional inductive CT having 50 Hz rated frequency can be modeled using the usual equivalent circuit reported in Figure 1; in this case, all the elements are reported to the secondary side. Parameters were measured on a real CT, and they are listed in Table 1; the rated secondary current is 5 A.

As aforementioned, accurate modeling of core nonlinearities has vital importance for this work. Therefore, the relationship between the magnetizing current *i_m_* and the mutual flux linkage *ψ_m_* was represented by using the Tellinen hysteresis model [33]. The symmetric, major hysteresis loop was measured under quasi-steady-state conditions so that the effect of eddy current is negligible. When the model is employed, the operating point of the core is assumed to be within the measured major loop. In turn, this was divided into its ascending and descending part so that two functions *ψ*_+_ (*i_m_*) and *ψ*_−_ (*i_m_*) were obtained, respectively. After this, the incremental inductances of the ascending and descending part (*L*_+_ (*i_m_*) and *L*_−_ (*i_m_*)) were computed. Using these results, the Tellinen model allows obtaining the derivative of the magnetizing current from *ψ_m_*; in turn, *i_m_* results from integration.

(14) $$\left\{ \begin{array}{l} \frac{di_{m}}{dt}=\frac{\psi_{-}\left(i_{m}\right)-\psi_{+}\left(i_{m}\right)}{\left[\psi_{-}\left(i_{m}\right)-\psi_{m}\right]L_{+}\left(i_{m}\right)}v_{m}\;v_{m}\geq 0\\ \frac{di_{m}}{dt}=\frac{\psi_{-}\left(i_{m}\right)-\psi_{+}\left(i_{m}\right)}{\left[\psi_{m}-\psi_{+}\left(i_{m}\right)\right]L_{-}\left(i_{m}\right)}v_{m}\;v_{m}<0 \end{array}\right.$$

In addition, the parallel resistor *R_m_* was introduced to model eddy current loss, thus supposed to be proportional to the square of the electromotive force *v_m_*. Its value can be obtained by matching the open-circuit loss predicted by the model at rated flux and 50 Hz sinusoidal excitation with the measured value.

### 3.2. Simulation Results

As aforementioned, the target of the numerical simulations is comparing the accuracies that can be achieved by the two proposed methods in reconstructing the primary current harmonics from the secondary side of the CT model. Compensation degrees *D* ranging from 1 (linear reconstruction) to 11 were considered. First, the coefficient vectors **K***_m_* and **H***_m_* must be estimated for each degree. According to the procedure described in Section 2, this requires applying a proper set of primary quasi-sinusoidal multisine current waveforms having a 50 Hz frequency, resembling those that can be found during regular operation; the fundamental amplitudes shall span the whole measurement range of the CT. In this respect, [10] states that metrological performance must be tested between 5% and 120% of the rated primary current. According to these considerations, a class *E*_1_ of current waveforms was defined. The fundamental component has a uniform probability density function (PDF) between 5% and 120% of the rated value. Superimposed harmonics up to order *M* = 31 are characterized by the same amplitude, which is 2.5% of that of the fundamental, thus corresponding to a total harmonic distortion (THD) level of 13.7%. Their phases were randomly extracted with independent, uniform PDFs between −*π* and *π*. *P* = 100 current waveforms belonging to the previously defined class were generated and applied to the reference model of the CT. The corresponding steady-state secondary currents were saved with a 200 kHz sampling rate, thus corresponding to *N_C_* = 4000 samples per period. In this way, harmonics can be extracted by using the discrete Fourier transform (DFT) without spectral leakage effects. After this, (9), (10), (13) were inverted with the LS approach so that the unknown coefficients were computed.

Achieved performance was assessed by applying to the CT model a new set of *P* = 2500 current waveforms belonging to the previously defined class *E*_1_ and evaluating the resulting secondary current harmonics. The proposed techniques were employed to reconstruct the primary current harmonics from the secondary current spectrum. To quantify the accuracy, for each *p*th validation signal, HD compensation method and degree *D*, the normalized root-mean-square error (NRMSE) was computed as:

(15) $${\mathrm{NRMSE}}^{\left[p\right]}=\sqrt{\frac{\sum_{n=0}^{N_{C}-1}{\left(i_{1,e}^{\left[p\right]}\left(nT_{S}\right)-i_{1}^{\left[p\right]}\left(nT_{S}\right)\right)}^{2}}{\sum_{n=0}^{N_{C}-1}{\left(i_{1}^{\left[p\right]}\left(nT_{S}\right)\right)}^{2}}}$$

where *T_s_* is the sampling interval, while *i*_1_(*nT_s_*) and *i*_1,*e*_(*nT_s_*) are the samples of the actual and reconstructed primary current, respectively. For better computational efficiency, the corresponding frequency-domain expression was adopted.

**Figure 2:**
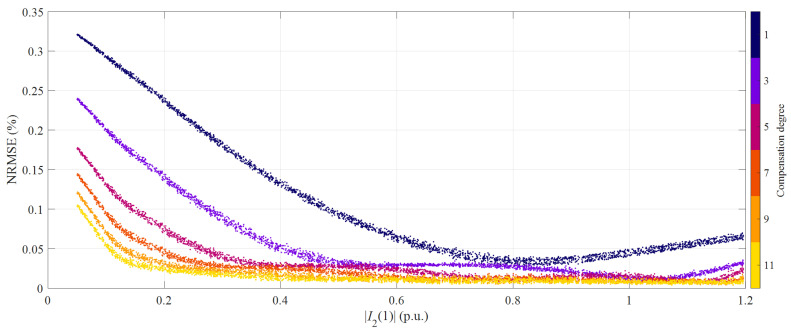
NRMSE as a function of the fundamental secondary current magnitude for different HD compensation degrees (numerical simulations).

The major difference between current and voltage measurements is that a considerably wider range is required in the former case. In general, measurement accuracy is strictly related to the magnitude of the measurand; in fact, for a given accuracy class, [10] specifies different maximum errors according to the primary current amplitude. For this reason, it is interesting to plot the obtained NRMSE values as a function of |*I*_2_(1)|. Results obtained with the full HD compensation method are reported in Figure 2, where the color denotes the degree. When adopting the simplified approach, performance is virtually unchanged: if the obtained NRMSE values were plot together in Figure 2 (not shown for the sake of clarity), they would be almost completely overlapped.

It is worth noting that only the results for odd degree compensations were reported: during simulations, symmetric hysteresis loops are described, thus producing purely odd-degree nonlinearities. Therefore, passing from an odd-order to the next even-order compensation does not increase accuracy. For the same reason, the HD compensation techniques are expected to improve only the measurement of odd-order harmonics.

As expected, accuracy is progressively enhanced as long as the order of the compensation method is increased. The highest NRMSE values always occur at lower amplitudes, whatever compensation degree is considered. On the other hand, [10] allows considerably larger errors at low amplitudes for a given accuracy class. Nevertheless, also at 5% of the rated amplitude, the proposed HD compensation methods are extremely effective since NRMSE reduces from 0.32% to 0.10%. The 11th order methods result in a plateau for currents between 40% and 120% of the rated value. This range will be carefully investigated in the following.

**Figure 3:**
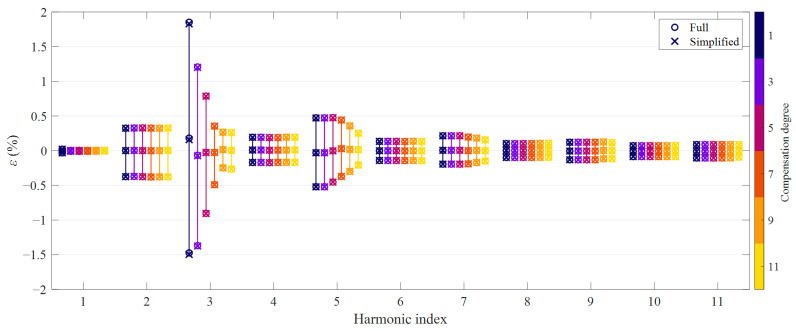
Ratio error achieved with the proposed HD compensation methods: odd degrees from 1 to 11, average values and 95th percentile bands (numerical simulations).

**Figure 4:**
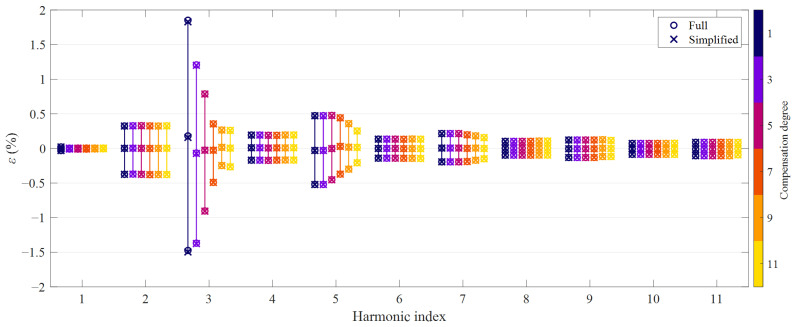
Phase displacement achieved with the proposed HD compensation methods: odd degrees from 1 to 11, average values and 95th percentile bands (numerical simulations).

The accuracy of CTs is conventionally quantified in terms of ratio error and phase displacement. In principle, these metrics can be used both for the fundamental and for the harmonic components, as for LPITs [11]. Hence, for a given *D*th degree HD compensation method, *m*th order harmonic, *p*th test signal, ratio error *ε*^[*p*]^(*m*) and phase displacement Δ*φ*^[*p*]^(*m*) are defined as:

(16) $$\begin{array}{c} \varepsilon^{\left[p\right]}\left(m\right)=\frac{\left|I_{1,e}^{\left[p\right]}\left(m\right)\right|-\left|I_{1}^{\left[p\right]}\left(m\right)\right|}{\left|I_{1}^{\left[p\right]}\left(m\right)\right|}\\ \Delta \varphi^{\left[p\right]}\left(m\right)=∠\left(I_{1,e}^{\left[p\right]}\left(m\right)\right)-∠\left(I_{1}^{\left[p\right]}\left(m\right)\right) \end{array}$$

where *I*_1_^[*p*]^(*m*) is the *m*th order harmonic in the *p*th primary current waveform, while *I*_1,*e*_^[*p*]^(*m*) is the corresponding reconstruction obtained with the proposed methods. For each harmonic order, compensation approach and degree, the average value was obtained; the dispersion was quantified by computing the 2.5th and 97.5th percentiles values. Figure 3 and Figure 4 summarize the results in terms of ratio and phase errors, respectively: circle markers denote the average value and the 95th percentile bands obtained with the full HD compensation approach, while cross markers correspond to the simplified method. Harmonics up to the 11th order are shown since it is the highest that may benefit from the 11th degree HD compensation, as explained in Section 2.

Similarly to NRMSE values, also the ratio errors and phase displacements obtained with the full and the simplified HD compensation approach almost overlap. Therefore, it is more interesting to analyze the results achieved with the different degrees. In general, the average values of the errors are very small; a noticeable bias occurs only for the magnitude error at the third-order harmonic as far as a linear reconstruction is employed. On the other hand, the HD compensation methods result in considerably narrower 95th percentile bands, namely a smaller dispersion of the magnitude error values; this reduction is particularly evident for the third and fifth-order harmonics.

Similar values can be found when looking at the 95th percentile bands of the phase displacements of the harmonics if angles are expressed in centiradians. In any case, residual errors are due to nonlinear effects that are not included in the compensation model, hence to higher-order HD and, most important, intermodulation between different spectral components in the primary current. Moreover, from Figure 3 and Figure 4, it is evident that, as expected, errors at even order harmonics are not mitigated by HD compensation.

From simulation results, it can be clearly noticed that considering this application, the full and the simplified HD compensation methods reach virtually the same accuracy. The reason is that assuming a linear relationship between primary and secondary fundamental current when deriving the compensation method introduces an approximation that is negligible when compared to the inherent definitional uncertainty of the underlying model, namely considering finite-degree HD as the only nonlinear effect. Therefore, thanks to the easier implementation, the simplified approach represents the most attractive method in this case; for this reason, the full HD compensation method will be no longer considered in the following.

## 4. Experimental Setup

**Figure 5:**
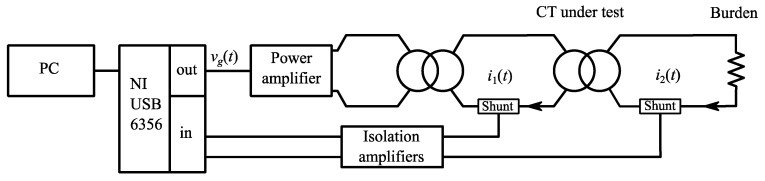
Block diagram of the experimental setup.

The simplified HD compensation technique was applied to improve the performance of conventional inductive CTs. As explained in the previous section, identifying the coefficients and validating the achieved performance requires applying complex multisine primary current waveforms; for this purpose, an arbitrary current waveform generator (ACWG) based on the architecture proposed in [34] was implemented. Its schematic diagram is shown in Figure 5.

The ACWG consists of a voltage waveform generator connected to a power amplifier (AE Techron 7548, specifications are reported in Table 2) that in turn feeds a transformer supplying the primary winding of the CT under test. The role of this interposed transformer is boosting the output current capability of the adopted power amplifier, thus permitting to test CTs having larger primary current ratings.

The input voltage of the power amplifier is obtained by proper prefiltering of the desired primary current excitation to compensate for the frequency response of the generation system; of course, its nonlinearities cannot be mitigated. However, thanks to the features of the proposed approach, the accuracy of the generation system is not critical for the achieved metrological performance: it just affects how much the class of the applied signals is similar to the desired one. Instead, having available precise measurements of the applied current spectrum and of the corresponding CT response plays a key role for both a proper identification and a significant evaluation of the achieved performance.

Primary and secondary currents were measured with two high-precision, class 0.2 coaxial shunts, both characterized by DC–20 kHz bandwidth and rated currents of 100 A and 10 A, respectively. Their outputs were connected to Analog Devices AD215BY isolation amplifiers operating in noninverting configuration with nominal gains equal to 100; their frequency responses were measured and digitally post-compensated in the frequency domain. After calibration, the current measurement channels have overall residual gain uncertainty below 100 ppm and phase uncertainty lower than 0.2 mrad in the range 0–5 kHz.

Data acquisition and generation of the voltage to be applied at the input of the power amplifier were provided by a National Instruments USB-6356 board with simultaneous sampling capability and a maximum rate of 1.25 Msamples/s per channel 16-bit resolution and adjustable voltage range. Input channels are characterized by total harmonic distortion lower than −80 dB, namely well below that is expected to be introduced by the CT under test. Otherwise, a significant evaluation of the achieved performance would not be possible. Signal generation, acquisition and processing were managed using a PC.

## 5. Experimental Results

The proposed HD compensation method was tested on two different inductive CTs having different accuracy classes. The current generator introduced in the previous section was employed to supply the CT under test with a set of *P* = 500 multisine primary currents belonging to class *E*_1_; 100 periods of the steady-state primary and secondary currents were acquired with a 200 kHz sampling rate. To improve the signal-to-noise ratio, their average spectra were computed. The experimental setup guarantees synchronized acquisition and generation processes. Therefore, spectral components obtained with the DFT are not affected by leakage artifacts. For each generic *m*th order harmonic and *D*th compensation degree (ranging from 1 to 11), the vector **K***_m_* can be estimated using the acquired data with the LS procedure described in Section 2. It is worth highlighting once again that since the applied current waveforms are acquired, and there are no specific constraints about the set of primary current waveforms used for the identification, the accuracy of the current generation system is not critical.

Once having identified the different compensation filters, they were employed to improve the measurement capabilities of the CT under test. For this purpose, it was supplied with a second set of *P* = 2000 primary currents belonging to the same class *E*_1_ as that used in the identification procedure.

### 5.1. Tests on CT1

The HD compensation technique was first tested on the inductive current transformer CT1, having a 50 Hz rated frequency. It has a window and a multi-tap primary winding so that the rated primary current can be set from 10 A to 600 A. During the tests, 50 A primary current was considered; the other specifications are summarized in Table 3. The secondary winding was connected to its rated, resistive burden.

**Figure 6:**
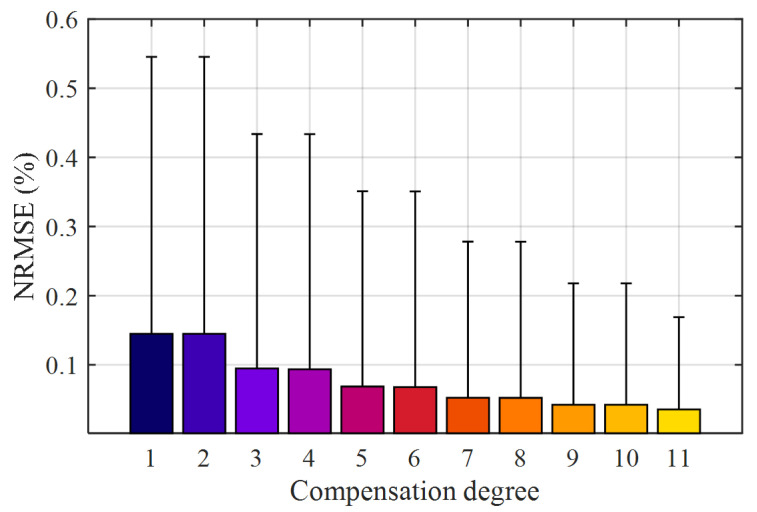
Average (bars) and 95th percentile values (error bars) of the NRMSE obtained with HD compensations of different degrees; transformer CT1, class *E*_1_ of primary current waveforms.

The primary current waveforms used for identification and validation belong to the same class *E*_1_ defined in Section 3 that was also employed in the numerical simulations. Achieved performance was first quantified in terms of NRMSE: its 95th percentile and average values are reported in Figure 6 for each considered HD compensation degree.

As expected, the experimental results show that both the 95th percentile and the average NRMSE values decrease as long as the order of the compensation filter is increased. In particular, when comparing the optimal linear reconstruction of the primary current with the 11th degree HD compensation, the 95th percentile NRMSE drops from 0.55% to 0.17%, while its mean value from 0.14% to 0.035%. As expected, most of the benefits are due to odd-order terms: in fact, by increasing the compensation degree from an odd one to the next even, performance is virtually unchanged. For this reason, from now on, only odd-degree HD compensation filters will be taken into account.

**Figure 7:**
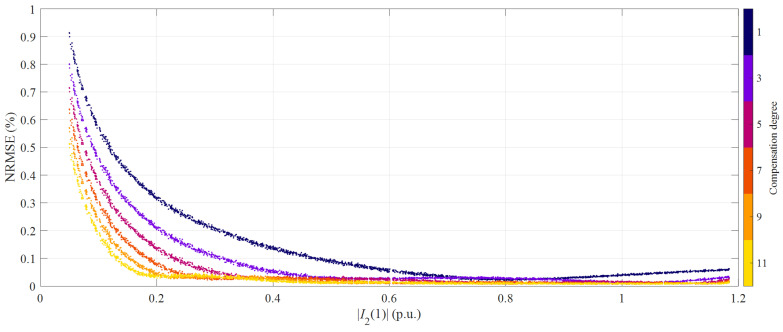
NRMSE as a function of the fundamental secondary current magnitude for different HD compensation degrees; transformer CT1, class *E*_1_ of primary current waveforms.

In Figure 7, the NRMSE is shown as a function of the magnitude of the fundamental secondary current. As observed in numerical simulations, the highest NRMSE values occur in the leftmost part of the graph, thus for the lowest currents (5% of the rated value). Furthermore, in this case, the proposed HD compensation allows almost halving the NRMSE, which drops from 0.9% to below 0.6%. It is worth observing that the reduction is considerably lower when compared to that expected from numerical simulations; the poor signal-to-noise ratio at small current amplitudes may play a significant role. On the other hand, considering the 11th degree HD compensation, NRMSE values are rather flat in the range between 40% and 120% of the rated current: in this respect, the trend is extremely similar to that predicted by simulations. Only the data belonging to this amplitude range will be analyzed in the following.

**Figure 8:**
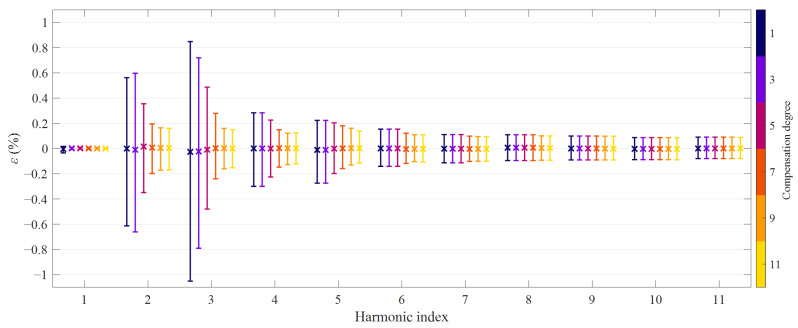
Ratio errors achieved with HD compensation degrees from 1 to 11: average values and 95th percentile bands; transformer CT1, class *E*_1_ of primary current waveforms.

**Figure 9:**
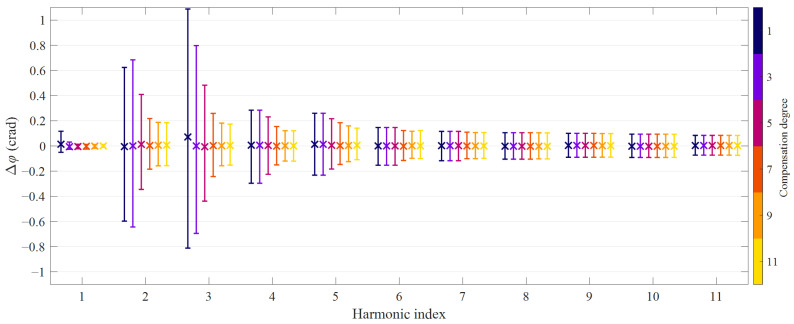
Phase displacement achieved with HD compensation degrees from 1 to 11: average values and 95th percentile bands; transformer CT1, class *E*_1_ of primary current waveforms.

The harmonic measurement performance achieved thanks to the proposed HD compensation method was quantified in terms of ratio error and phase displacement. As in the numerical simulations, they were computed for each excitation signal, compensation degree and harmonic order. Their average, 2.5th and 97.5th percentile values were calculated over the *P* test realizations. Results are shown in Figure 8 and Figure 9: cross markers represent the average values, while error bars define the 95th percentile bands.

Let us start analyzing the magnitude error. When looking at the average values, it can be concluded that errors are virtually unbiased, except for the linear reconstruction of the third-order harmonic (0.07%), similarly to the simulation results. At low-order, odd harmonics the spread of the ratio error decreases dramatically as long as the HD compensation is employed. For example, the width of the 95th percentile band decreases from 1.8% to 0.32% at the 3rd order harmonic, showing a behavior that is similar to what was observed in numerical simulations. However, it should be noticed that experimental data denote a relevant ratio error also at even harmonics, in particular at the second-order one. The reason is remanence (considered to be zero during simulations), which makes the hysteresis loops no longer symmetric, thus producing also even degree nonlinearities. In any case, the proposed HD compensation can considerably reduce this effect, as highlighted by Figure 8: ratio error at the second-order harmonic decreases to 0.32%, thus close to what is achieved for the other components.

According to simulations, phase and ratio errors at the harmonics exhibit similar values when expressed in centiradians and percentage, respectively; unsurprisingly, the same considerations apply also for experimental results. It is clear that ratio error and phase displacement at each harmonic are strongly correlated: the reason is the combination between the nonlinearity of the device and the choice of the class of test signals [35].

To further investigate the robustness of the proposed method, it was validated by considering a new class *E*_2_ of multisine primary currents waveforms. Like the previously defined class *E*_1_, it is still characterized by 50 Hz fundamental frequency having random fundamental amplitude with a uniform distribution between 40% and 120% of its rated value. However, the harmonic amplitudes of the signals belonging to *E*_2_ are characterized by uniform PDFs in the range of 0.5–5% of the fundamental. This corresponded to an expected THD value of 15.1% and a maximum of 27.4%. Harmonic phases have uniform distributions between −*π* and *π*. *P* = 1000 random primary current waveforms were extracted by sampling the previously defined PDFs and then applied to CT1.

The achieved improvement in harmonic measurement accuracy that can be obtained with the proposed technique was quantified in terms of total vector error (TVE), which is capable of taking into account both ratio error and phase displacement simultaneously. On the other hand, previous graphs show that their values at the harmonics are strongly correlated. For each *p*th validation signal, *m*th harmonic order and *D*th HD compensation degree, the TVE represents the distance in the complex plane between the actual primary current phasor and the reconstructed one. The TVE can be expressed as a percentage of the actual current phasor, thus:

(17) $${\mathrm{TVE}}_{m}^{\left[p\right]}\left(m\right)=\frac{\left|I_{1,e}^{\left[p\right]}\left(m\right)-I_{1}^{\left[p\right]}\left(m\right)\right|}{\left|I_{1}^{\left[p\right]}\left(m\right)\right|}$$

**Figure 10:**
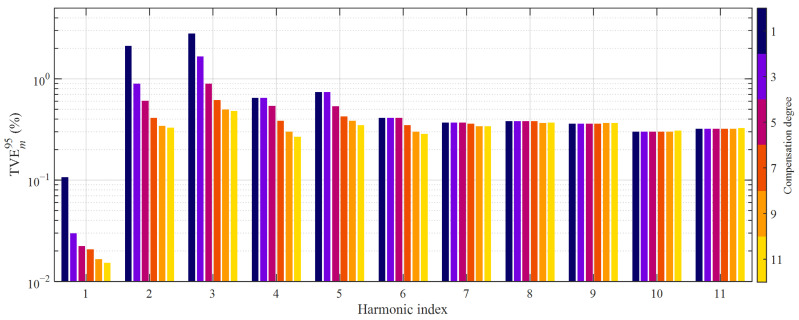
95th percentile values of TVE achieved with HD compensation degrees from 1 to 11; transformer CT1, class *E*_2_ of primary current waveforms.

The 95th percentile values of the TVE over the *P* test realizations were computed for each harmonic order and HD compensation degree; results are summarized in Figure 10.

Errors were generally higher than those obtained by considering the previous class *E*_1_ of primary current waveforms; when analyzing the data, it can be concluded that the highest TVE values occur as long as the harmonics to be measured have the smallest relative amplitudes. This is somewhat expected in this case: as from (2), the HD produced by the fundamental component has the maximum relative impact in this case. Conversely, the lowest errors are achieved when the harmonic to be measured larger. In any case, the enhancement obtained with HD compensation was evident in the left part of the spectrum. For example, TVE*_m_*^95^ decreased from 2.81% to 0.48% for the 3rd order harmonic. A remarkable improvement was also achieved at the 2nd order harmonic, since the 95th percentile value of the TVE dropped from 2.1% to 0.3%. Results for the fundamental component are also reported; it suffered just from weak nonlinear effects. but TVE*_m_*^95^ is reduced from 0.11% to 0.015%, thanks to the proposed approach. This nonlinearity at the fundamental component is generally much less noticeable in VTs because of their considerably narrower measurement range.

As mentioned at the beginning of the subsection, CT1 can be connected to obtain several rated primary current values; basically, the number of primary turns is changed so that all the possible rated currents correspond to the same magnetomotive force value. Therefore, very similar harmonic measurement performance is expected for all the configurations, since the magnetic effect of the primary current just depends on its per-unit value.

As a final remark, it should be mentioned that, as previously stated, the nonlinear behavior of the CT was somewhat dependent on the remanent magnetization of its core, which was affected by faults and transients. Therefore, the effectiveness of the technique may be reduced in this case, in particular at even-order harmonics and for small current amplitudes. However, this is a common issue for all the post-compensation techniques for inductive CTs, since the magnetization state of the core cannot be observed from the secondary current.

### 5.2. Tests on CT2

The proposed HD compensation technique was also tested on a second inductive current transformer CT2, still characterized by 50 Hz rated frequency but having different specifications, which are summarized in Table 4. Also in this case, the secondary winding was connected to its rated resistive burden.

**Figure 11:**
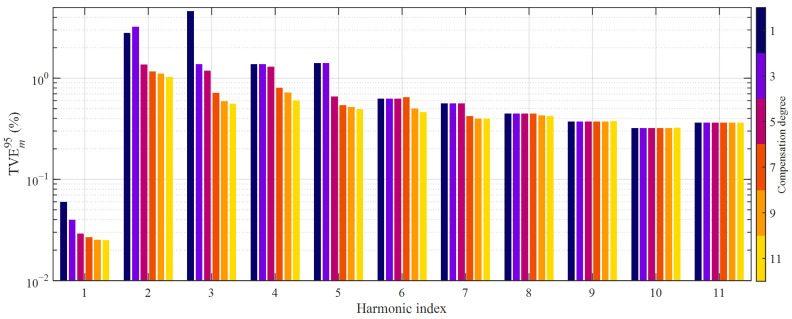
95th percentile values of TVE achieved with HD compensation degrees from 1 to 11; transformer CT2, class *E*_2_ of primary current waveforms.

The parameters of the HD compensation filters were estimated with the same procedure as that followed for CT1. However, metrological performance was evaluated by applying a set of *P* = 1000 multisine primary current waveforms belonging to the class *E*_2_ introduced at the end of the previous subsection. For the sake of brevity, accuracy for each harmonic order *m* and compensation degree *D* was evaluated only in terms of the 95th percentile value of the TVE over the *P* validation signals. Figure 11 summarizes the obtained results.

It is worth reminding that CT1 is characterized by accuracy class 0.5, while CT2 has higher rated metrological performance, being it class 0.2. When comparing Figure 10 with Figure 11, it is not surprising that considering the optimal linear reconstruction of the fundamental primary current, CT2 reaches a significantly lower 95th percentile value of TVE, 0.06% concerning 0.11%. However, when introducing the HD compensation, CT1 reaches better overall accuracy. Furthermore, considering the linear reconstruction of the other harmonics, CT1 is considerably more precise despite its worse rated accuracy class. Nevertheless, the proposed HD compensation method proves to be highly effective also when it is applied to CT2: for example, the 95th percentile TVE value at the third-order harmonic is lowered by more than 7 times: it falls from over 4.6% (linear reconstruction) to 0.56% (11th degree HD compensation). Remarkable accuracy improvement is clearly noticeable also at the second, fourth and fifth-order harmonics. It is worth noting that when comparing the results obtained thanks to HD compensation, CT1 still achieves significantly better metrological performance than CT2.

## 6. Conclusions

Harmonic current measurements are often performed by using inductive CTs, even if it is well-known that their rated metrological performance is guaranteed only at the fundamental. In general, for the other components, the achieved accuracy is considerably worse than that expressed by their accuracy class because of two effects: their filtering behavior and the nonlinearities introduced by their iron cores. In this respect, the strongest nonlinear phenomenon is the HD produced by the strong fundamental component that significantly affects the low-order harmonics at the secondary side. In the present paper, a method that allows compensating for the HD and the CT’s filtering behavior was proposed and experimentally validated on two specimens having different accuracy classes. Based on polynomial modeling of HD, the technique is characterized by easy implementation and low computational complexity. The LS-based identification procedure permits high flexibility in choosing the primary current waveforms required for estimating the parameters. For the very same reason, the method is also robust concerning the accuracy of the generation system employed to apply these currents, thus reducing the cost of the equipment required for the implementation. The obtained results highlight a remarkable accuracy improvement over a wide range of current amplitudes, particularly at low-order harmonics. It is worth stressing that the achieved performance is not related to the accuracy class: in fact, the best results are obtained with the CT characterized by the worst rated accuracy. Thanks to the black-box approach, the technique can also be applied to low-power CTs based on different operating principles. These features make the proposed method an attractive solution to be implemented in measuring instruments, such as power quality analyzers and merging units. For best performance, each CT must be provided with its specific compensation parameters. This means that each specimen must be characterized. As future work, it is worth investigating whether it is possible to adopt the same compensation parameters for nominally identical CTs and quantifying the resulting performance degradation.

## Figures and Tables

**Table 1:** Parameters of the equivalent circuit.

*R_m_* (Ω)	*R*_2_ (Ω)	*L_l_* (μH)	*R_L_* (Ω)
250	0.1	43	0.4

**Table 2:** Power amplifier specifications.

*V_max_* (V)	*I_max_* (A)	Frequency Response	THD	SNR (dB)	Voltage Gain
200	43	0–30 kHz +0.1, −0.5 dB	<0.1% DC–30 kHz	>120	20

**Table 3:** Current transformer CT1: rated specifications.

*I*_1*n*_ (A)	*I*_2*n*_ (A)	Class	Burden (VA)
50	5	0.5	10

**Table 4:** Current transformer CT2: rated specifications.

*I*_1*n*_ (A)	*I*_2*n*_ (A)	Class	Burden (VA)
10	5	0.2	5
